# Blood Transfusion in End-of-Life Cancer Care: Clinical Evidence, Patient Blood Management and Goal-Concordant Practice

**DOI:** 10.3390/medsci14050512

**Published:** 2026-08-25

**Authors:** Saikat Mandal, Manideepa Maji, Ashish Sharma, Arkadeep Dhali

**Affiliations:** 1 Translational Medical Sciences, School of Medicine, University of Nottingham, Nottingham NG7 2UH, UK; saikat.mandal@nottingham.ac.uk; 2 NIHR Nottingham Biomedical Research Centre, Nottingham University Hospitals NHS Trust, Nottingham NG7 2UH, UK; 3 Hull York Medical School, University of Hull, Hull HU6 7RX, UK; manideepa.maji@hyms.ac.uk; 4 Haematology, Hull University Teaching Hospitals NHS Trust, Hull HU16 5JQ, UK; 5 Medical Oncology, Smilow Cancer Hospital, Yale New Haven Hospital, New Haven, CT 06510, USA; ashish.sharma@ynhh.org; 6 Sheffield Teaching Hospitals NHS Trust, Sheffield S5 7AU, UK; 7 Division of Surgery and Cancer, Imperial College London, London W12 0NN, UK; 8 School of Medicine, Dentistry and Biomedical Sciences, Queen’s University Belfast, Belfast BT9 7BL, UK

**Keywords:** blood transfusion, end-of-life care, palliative care, red blood cells, platelets, hospice

## Abstract

Blood transfusion near the end of life for patients with cancer may relieve symptoms attributed to anaemia or control bleeding, but its palliative value depends on whether benefit occurs within the patient’s expected survival and outweighs the clinical and practical burden of treatment. This structured narrative review examines evidence concerning red-cell and platelet transfusion, transfusion-sparing patient blood management, hospice access and alternative delivery models in adults receiving palliative or end-of-life care. Structured searches of MEDLINE, Embase, Scopus and the Cochrane Library identified original studies, audits, qualitative research and relevant evidence syntheses. The evidence was predominantly observational and methodologically heterogeneous. Reported symptomatic response rates after red-cell transfusion ranged from 31% to 70%, most commonly involving short-term improvement in fatigue, dyspnoea or general well-being. Benefit often diminished within 14 days, while 23–35% of participants in historical cohorts died within two weeks, limiting the opportunity to experience benefit. Response was not reliably predicted by haemoglobin concentration. Red-cell transfusions were frequently guided by laboratory values, administered late in the disease course and not followed by systematic reassessment. Evidence concerning platelet transfusion remains largely descriptive and does not establish a prophylactic threshold for end-of-life care. Intravenous iron and haemostatic radiotherapy may reduce transfusion requirements in selected patients, although their applicability depends on clinical stability, the source of bleeding and sufficient time to benefit. In haematological malignancies, transfusion dependence has been associated with lower hospice enrolment and shorter hospice stays, whereas home- and hospice-based transfusion may reduce travel and waiting burdens for appropriately selected patients. Transfusion near the end of life should therefore be considered a goal-concordant, time-limited trial. A patient-valued outcome should be defined beforehand, the fewest units likely to achieve that outcome should be administered, and benefit should be reassessed using the same measure. Further transfusion should be offered only when documented symptomatic or functional benefit outweighs adverse effects and treatment burden for the individual patient.

## 1. Introduction

Palliative care may be introduced at any stage of a serious illness and provided alongside disease-modifying treatment. As the illness progresses and such disease-modifying treatment becomes ineffective, poorly tolerated or inconsistent with the patient’s priorities, the emphasis of care increasingly shifts towards symptom relief, comfort, preservation of function and preferred place of care. End-of-life care represents a later phase within this broader palliative-care continuum, although its duration varies according to the underlying disease and clinical trajectory. Hospice is a model of predominantly comfort-focused care governed by local eligibility and funding arrangements, whereas active dying denotes the final phase of irreversible clinical deterioration. These distinctions are particularly important when considering transfusion. A patient with transfusion-dependent marrow failure or terminal cancer may remain clinically stable for months and have time to benefit, whereas a patient with multiorgan failure may have only days to live. The value of transfusion must therefore be assessed against the expected prognosis, time to benefit and the patient’s goals of care [1,2,3].

Blood components have established supportive roles in oncology and haematology. Red-cell transfusion increases oxygen-carrying capacity, whereas platelets, plasma and cryoprecipitate may be used for defined bleeding or haemostatic indications. During disease-modifying treatment, transfusion may also support the delivery of chemotherapy or an invasive procedure. However, when the primary goal of care shifts towards palliation, the indication for transfusion should be carefully reassessed. Correction of an abnormal blood count alone is insufficient; transfusion should be offered only when it is expected to improve comfort, function or another patient-valued outcome [4,5].

Anaemia and thrombocytopenia are common in advanced solid tumours and haematological malignancies due to inflammation, marrow infiltration or failure, bleeding, renal impairment, nutritional deficiency, and previous treatment. Fatigue, weakness and dyspnoea, however, may also arise from infection, cachexia, cardiopulmonary disease, pain, medication, anxiety or deconditioning. A blood count is therefore an aid to assessment, not a palliative outcome: neither low haemoglobin nor its post-transfusion increment reliably predicts symptom relief. Some patients benefit, but the response is difficult to predict and usually brief [4,5,6,7,8,9,10,11,12,13]. Practice also varies; symptom-led intent may coexist with haemoglobin triggers and unstructured reassessment [14]. Evidence for platelet transfusion near death is even more limited, particularly for prophylaxis [15].

Blood sampling, compatibility testing, travel, waiting, infusion and observation may occupy a substantial part of the patient’s remaining time. In leukaemia, myelodysplastic syndromes and other marrow-failure disorders, recurrent transfusion may also maintain reliance on hospital care. This does not imply inappropriate treatment, but population studies associate transfusion dependence with lower hospice enrolment or shorter stays, and surveys indicate that access influences care decisions [16,17,18,19]. Repeated attendance can increase caregiver workload and make remaining at home more difficult. Conversely, stopping transfusion without a shared plan may complicate symptom relief or functional independence for patients who have previously benefited.

Transfusion near the end of life should therefore be neither a routine response to an abnormal blood count nor an intervention withdrawn solely because prognosis is limited. This review examines symptom response and its duration, platelet support, adverse effects, patient blood management, hospice access and alternative delivery models. Its central question is how blood components can be used when they advance a patient-valued goal and discontinued when they no longer do so.

## 2. Methodology

This structured narrative review was informed by a structured literature search and developed with reference to the Scale for the Assessment of Narrative Review Articles (SANRA) [20]. MEDLINE via PubMed, Embase, Scopus, and the Cochrane Library were searched from database inception to 12 July 2026, with no publication-date restriction. Database-specific subject headings and free-text terms were combined for two concepts: blood transfusion, end-of-life care and advanced cancer.

The search strategy was: (“Blood Transfusion”[MeSH] OR “Erythrocyte Transfusion”[MeSH] OR “Platelet Transfusion”[MeSH] OR transfus*[Title/Abstract] OR “red blood cell”[Title/Abstract] OR “red blood cells”[Title/Abstract] OR “red cell”[Title/Abstract] OR “red cells”[Title/Abstract] OR platelet*[Title/Abstract] OR “blood component”[Title/Abstract] OR “blood components”[Title/Abstract] OR “transfusion dependence”[Title/Abstract] OR “transfusion dependent”[Title/Abstract]) AND (“Palliative Care”[MeSH] OR “Hospice Care”[MeSH] OR “Terminal Care”[MeSH] OR palliative[Title/Abstract] OR hospice*[Title/Abstract] OR “end of life”[Title/Abstract] OR “end-of-life”[Title/Abstract] OR “terminal care”[Title/Abstract] OR “terminally ill”[Title/Abstract] OR “actively dying”[Title/Abstract]) AND (“Neoplasms”[MeSH] OR “Hematologic Neoplasms”[MeSH] OR cancer*[Title/Abstract] OR neoplasm*[Title/Abstract] OR malignan*[Title/Abstract] OR leukemia[Title/Abstract] OR leukaemia[Title/Abstract] OR “myelodysplastic syndrome”[Title/Abstract] OR “myelodysplastic syndromes”[Title/Abstract] OR “marrow failure”[Title/Abstract]). The syntax was adapted for the other databases. Supplementary searches combined the palliative-care and malignancy concepts with patient blood management, iron deficiency, intravenous iron, haemostatic radiotherapy, tumour bleeding, antifibrinolytics, endoscopic haemostasis and embolisation. Reference lists of relevant studies and professional guidance were also screened.

Eligible publications included prospective and retrospective studies, audits, cross-sectional and qualitative research, and systematic reviews or meta-analyses of transfusion or transfusion-sparing interventions in palliative, hospice or end-of-life care. Adult and predominantly adult mixed-age cohorts were eligible; paediatric-only studies were excluded. Guidelines, professional statements and narrative reviews informed context and interpretation but were not used to establish effectiveness. Commentaries, editorials, isolated case reports, non-human studies and publications without clinically relevant outcomes were excluded. Outcomes of interest were symptom response, bleeding, function, quality of life, survival, adverse events, treatment burden, component use, hospice access, patient blood management, alternatives to transfusion, place of care, patient or caregiver experience, and delivery models. Records were screened by title and abstract and, when relevant, by full text; reference lists were examined for additional studies. Study selection was purposive and iterative, as appropriate for a narrative review of a broad clinical question. The evidence was organised and discussed according to its main clinical themes: transfusion effectiveness and duration of benefit, platelet support, adverse effects, patient preferences, hospice access, patient blood management and models of transfusion delivery. Study design, selection bias, attrition, outcome measurement and confounding were considered when interpreting the findings. The search identified 1243 records. Following title, abstract and full-text screening, 51 publications were included in this structured narrative review: 26 directly examined transfusion in end-of-life, terminal or hospice populations or reported outcomes anchored to death; 12 examined broader palliative or advanced-disease populations; and 13 provided indirect evidence on transfusion-sparing interventions or alternative delivery models [4,5,6,7,8,9,10,11,12,13,14,15,16,17,18,19,21,22,23,24,25,26,27,28,29,30,31,32,33,34,35,36,37,38,39,40,41,42,43,44,45,46,47,48,49,50,51,52,53,54,55]. Because of substantial clinical and methodological heterogeneity, neither meta-analysis nor formal certainty-of-evidence assessment was undertaken.

The studies were not treated as methodologically equivalent. Prospective studies with prespecified symptom assessment informed clinical benefit; retrospective records and audits described practice, and surveys or qualitative studies examined experience. Response proportions were not pooled because populations, transfusion intent, assessors, timing and completeness of follow-up differed; Table 1 and Table 2 report these features alongside the findings.

## 3. Discussion

### 3.1. Clinical Effectiveness and Duration of Benefit

Restrictive transfusion thresholds guide practice in stable adults, but they cannot determine whether transfusion will achieve a palliative goal. Haemoglobin should be interpreted alongside the likely cause of symptoms, active bleeding, cardiopulmonary reserve, previous response, prognosis, time to benefit, risk of volume overload and the patient’s priorities. Transfusion should not be used simply to test whether multifactorial fatigue is caused by anaemia. When attribution is uncertain, a time-limited trial with the same outcome assessed before and after treatment is preferable to repeated transfusion triggered by the blood count alone [4,5,9].

No randomised trial has established the symptomatic effectiveness of red-cell transfusion in end-of-life cancer care. The 2012 Cochrane review of 12 before-and-after studies involving 653 participants reported subjective response rates of 31–70%; fatigue and breathlessness often improved initially, but benefit commonly waned by day 14, when 23–35% of participants had died [5]. A later systematic review of 13 observational reports comprising 3473 patients, 995 of whom were transfused, likewise found short-term improvement in most symptom studies but little information on duration, predictors or harms [4]. These estimates should not be interpreted as a pooled treatment effect.

Early observational studies generally reported short-term improvement in approximately 50–75% of assessed patients, but follow-up was incomplete, and outcome assessment ranged from patient report to clinician or staff judgement [6,7,8,12,13,21,22,23]. Attrition was substantial: Laird et al. documented an outcome for 38 of 100 episodes [8], while Brown et al. analysed 30 of 48 enrolled participants [13]. Because patients who died or became too unwell were often unavailable for reassessment, response rates calculated among evaluable survivors may overestimate benefit.

The largest prospective cohort enrolled 141 patients across 17 palliative-care sites. At day 7, clinicians recorded improvement in the predefined target symptom after 49% of evaluable episodes, compared with improvement in any assessed symptom after 78%; only 101 participants contributed benefit data [9]. A non-randomised study from Nepal found improvement in fatigue in both transfused and non-transfused groups, leaving the treatment effect uncertain [24]. Later retrospective studies documented benefit after 25–36% of all episodes, but outcomes were missing after up to 60%, and better performance status rather than pre-transfusion haemoglobin was associated with response [25,26].

Studies of transfusion practice indicate that transfusions near the end of life are often prompted by laboratory values rather than symptoms, while clinical benefit is not consistently reassessed after transfusion [10,11,14,27]. In the national UK hospice audit, improvement persisted to day 30 after 18% of episodes, while 32% of patients died within 30 days [10]. Other cohorts reported median survival of 15.5–18 days and often lacked a recorded outcome [11,26]. Lack of documentation is not equivalent to confirmed non-response, but it prevents reliable evaluation.

Overall, short-term benefit is plausible, but its frequency and predictors remain uncertain. Before transfusion, the target symptom or functional goal and assessment time should be specified; the same measure should be reassessed within 48–72 h and before repeat transfusion. Effectiveness and practice evidence are summarised in Table 1 and Table 2.

### 3.2. Survival Is Not the Primary Palliative Outcome

Survival is relevant when transfusion is used to treat an acute, reversible and life-threatening anaemic or bleeding event, but it is not an appropriate primary endpoint for routine palliative transfusion. Retrospective comparisons are susceptible to confounding by indication, selection bias and immortal-time bias. Goksu et al. reported a median survival from final admission of 15 days among transfused patients and eight days among non-transfused patients [28]. In another study of 6980 patients referred for inpatient palliative-care consultation, 885 (12.7%) subsequently received red-cell transfusion. Transfused patients were younger, had better performance status and lower haemoglobin concentrations, and were more likely to have cancer. Median survival from the first transfusion was 56 days. Among patients who survived for at least 30 days after palliative-care consultation, transfusion was associated with greater odds of dying in hospital rather than at home, in long-term care or in a subacute-care setting (adjusted odds ratio 2.15, 95% confidence interval 1.71–2.70) [29]. These associations are vulnerable to residual confounding and cannot establish whether transfusion itself affected survival or place of death.

As summarised in Table 2, transfusions were frequently administered during the final days or weeks of life, limiting the time available to experience meaningful symptomatic or functional benefit. Death shortly after transfusion does not itself establish ineffectiveness, because short-term symptom relief may occur without prolonging survival. Nevertheless, proximity to death should prompt careful consideration of whether the likely benefit justifies the burden of transfusion.

### 3.3. Platelet Transfusion and Bleeding

Evidence on platelet transfusion near the end of life is limited and largely descriptive. In a cohort of 119 patients with haematological malignancies who received 1999 platelet concentrates during their final six months, 55% of transfusions were prophylactic, and 18% were administered during the final two weeks of life. Platelet increments and intervals between transfusions declined as death approached [15]. Other studies similarly report frequent prophylactic transfusion but provide limited component-specific data on bleeding control or clinical benefit [28,30,31].

In the absence of end-of-life-specific evidence, general thresholds may provide clinical reference points. NICE recommends prophylactic platelet transfusion at a platelet count below 10 × 10^9^/L in non-bleeding patients, except those with chronic bone marrow failure. For clinically significant bleeding, transfusion is recommended below 30 × 10^9^/L, while thresholds above 50 × 10^9^/L are advised for most invasive procedures and above 100 × 10^9^/L for surgery at critical sites [56]. These thresholds were not developed for end-of-life care and should not be applied as automatic transfusion triggers. Decisions should instead reflect the purpose of transfusion: prophylaxis, preparation for a procedure or treatment of active bleeding, together with prognosis, previous response, treatment burden and the patient’s goals.

### 3.4. Transfusion Dependence, Hospice Access and Patient Preferences

Observational studies, including large population-based analyses, associate transfusion dependence with lower hospice enrolment in myelodysplastic syndromes [16] and shorter hospice stays in leukaemia [17]. In other cohorts of patients with haematological malignancies, transfusions were frequently administered near the end of life, and significant number of patients died in acute-care hospitals [32,34,35]. Among 6955 older adults with myelodysplastic syndromes, transfusion dependence was associated with lower odds of hospice enrolment (adjusted odds ratio 0.69, 95% confidence interval 0.61–0.78) [16]. Similarly, among 21,033 patients with leukaemia, those who were transfusion-dependent had a median hospice stay of six days, compared with 11 days for non-dependent patients; after adjustment, their hospice stay was 51% shorter [17]. These observational findings do not establish that transfusion itself delays hospice referral or admission. More advanced disease, continued disease-directed treatment and the limited capacity of some hospices to provide transfusions may all contribute to these associations.

The availability of transfusion within hospice care is nevertheless important to patients. In a survey of 200 patients with haematological malignancies, transfusion was the most highly valued potential hospice service [18]. However, more than half of 113 US hospices reported that they never provided transfusions, and approximately three-quarters considered the lack of transfusion support a barrier to enrolment [19]. Qualitative studies suggest that decisions about continued transfusion are influenced by patients’ understanding of their prognosis, expectations that transfusion may relieve symptoms or preserve function, relationships with their existing clinical teams, concerns about adverse reactions, and preferences regarding place of care [36,37]. For patients who remain transfusion-dependent, the treating haematology or oncology team should initiate early discussions with the patient and, where appropriate, their family or carers. These discussions should involve the palliative-care team and address prognosis, the likely benefits and burdens of continued transfusion, preferred place of care, and the availability of transfusion support in hospice or community settings. Ongoing transfusion should not, by itself, delay referral to palliative or hospice care.

### 3.5. Adverse Effects and Treatment Burden

Immediate risks include acute haemolytic, febrile non-haemolytic and allergic reactions, bacterial contamination, transfusion-related acute lung injury and transfusion-associated circulatory overload. Overload is particularly relevant in frail patients, those with low body mass and those with cardiac or renal impairment, especially when multiple units are given without reassessment. Alloimmunisation can complicate compatibility and prolong waiting; iron accumulation remains relevant when prognosis is measured in months or years. Safety is poorly characterised because adverse-event reporting was sparse in the studies [4] and later cohorts used different definitions and surveillance methods [23,24,25]. Treatment burden also includes sampling, crossmatching, transport, waiting, infusion, observation and caregiver time. A short-lived response may have little overall value if much of it is spent arranging or receiving the next transfusion.

### 3.6. Patient Blood Management and Alternatives to Recurrent Transfusion

Intravenous iron may be useful when absolute or functional iron deficiency is documented or strongly suspected, marrow response remains possible, and the patient is likely to live long enough to benefit. Its potential advantages are gradual haemoglobin improvement and avoidance of some subsequent red-cell transfusions without transfusion-associated volume load or alloexposure. In a 34-patient feasibility trial, ferric derisomaltose resolved anaemia by eight weeks in 39% compared with 8% after placebo; smaller retrospective and feasibility studies suggested haemoglobin or quality-of-life improvement, but one excluded patients who died within 28 days, and none established durable symptomatic benefit or transfusion avoidance [38,39,40]. Intravenous iron does not provide immediate oxygen-carrying capacity and should not delay red-cell transfusion for severe symptomatic anaemia or active bleeding.

Benefit must be balanced against infusion reactions, uncertain infection risk and the practical burden of investigation and infusion. Across randomised trials in cancer-associated anaemia, infections were numerically more frequent after intravenous iron, but only eight of 18 trials reported infection and heterogeneity limited firm inference [48]. In predominantly perioperative gastrointestinal-cancer populations, intravenous iron was not associated with a statistically significant increase in infection (risk ratio 1.07, 95% confidence interval 0.71–1.62), although the estimate was imprecise and its applicability to end-of-life care is uncertain [47]. Broader evidence likewise does not establish safety during active infection [46]. The balance is most favourable in a clinically stable patient with iron-restricted erythropoiesis, anticipated survival of weeks to months and a wish to avoid recurrent transfusion; it is unlikely to be favourable when prognosis is very short or rapid symptom relief is required.

Other transfusion-sparing measures depend on the cause of anaemia or bleeding. Palliative radiotherapy in haemostatic indications may control focal tumour bleeding and reduce subsequent transfusion requirements, but the evidence is uncontrolled, tumour-site-specific and sensitive to early death [41,42,43]. A three-case report of heparin plus tranexamic acid for enhanced-fibrinolytic disseminated intravascular coagulation cannot guide routine care [44]. For stable, non-bleeding adults, single-unit red-cell transfusion followed by clinical reassessment remains a proportionate default [56,57]. Liberal thresholds may improve short-term quality of life in selected patients with non-curative myelodysplastic syndromes but require more units and have imprecise safety evidence [45]. The principal patient blood management evidence is summarised in Table 3; its application should remain individualised to prognosis and the patient’s goals [58].

### 3.7. Delivery Models, Ethics and Goal-Concordant Care

Hospital transfusion offers immediate laboratory and emergency support but requires travel and waiting. A review of 14 home-transfusion studies reported six severe reactions among 11,928 transfusions (0.05%), no transfusion-related deaths and no comparative effectiveness study [49]. A Japanese programme reported 1664 red-cell units given to 77 patients; there were no severe reactions and two mild allergic reactions. Unblinded next-day assessments recorded improvement in fatigue in 94.1% of patients with haematological malignancies and 53.8% with solid tumours, but major cardiac or renal risk was an exclusion criterion, and there was no comparator [50]. Other programmes and qualitative studies support feasibility while emphasising cold-chain control, emergency cover and caregiver confidence [51,52]. Patients receiving transfusions in a day hospice reported several advantages over hospital-based care, including easier transport, shorter waiting times and more opportunity to discuss their care with healthcare staff [53]. In a French cancer network, 63% of surveyed patients preferred future transfusions to be administered at home rather than in a hospital. Preference was greater among patients living farther from the hospital, those with poorer quality of life and those who had previously received home care [54]. However, a separate French service evaluation found that only three of six patients considered for home transfusion received it, largely because of clinical eligibility requirements and regulatory constraints concerning nursing responsibilities and physician availability [55]. These findings support non-hospital transfusion for selected patients but do not establish equivalence to hospital care. Safe pathways require compatibility testing, traceability, trained staff, observation, reaction management and rapid escalation.

Transfusion should be judged by the same ethical principles as other palliative interventions. Beneficence supports treatment likely to relieve a patient-valued problem; non-maleficence requires attention to reactions, circulatory overload and treatment burden; autonomy depends on realistic communication; and justice requires responsible use of a finite, donated resource. Limited prognosis alone is not a defensible reason for exclusion, but patient preference does not oblige clinicians to provide treatment when meaningful benefit is very unlikely. Funding should not force a choice between transfusion and hospice. Professional policy supports access to symptom-based palliative transfusion within hospice [59]. Recent analyses place the problem at the intersection of clinical uncertainty, resource allocation, reimbursement and hospice design [60,61].

**Figure 1:**
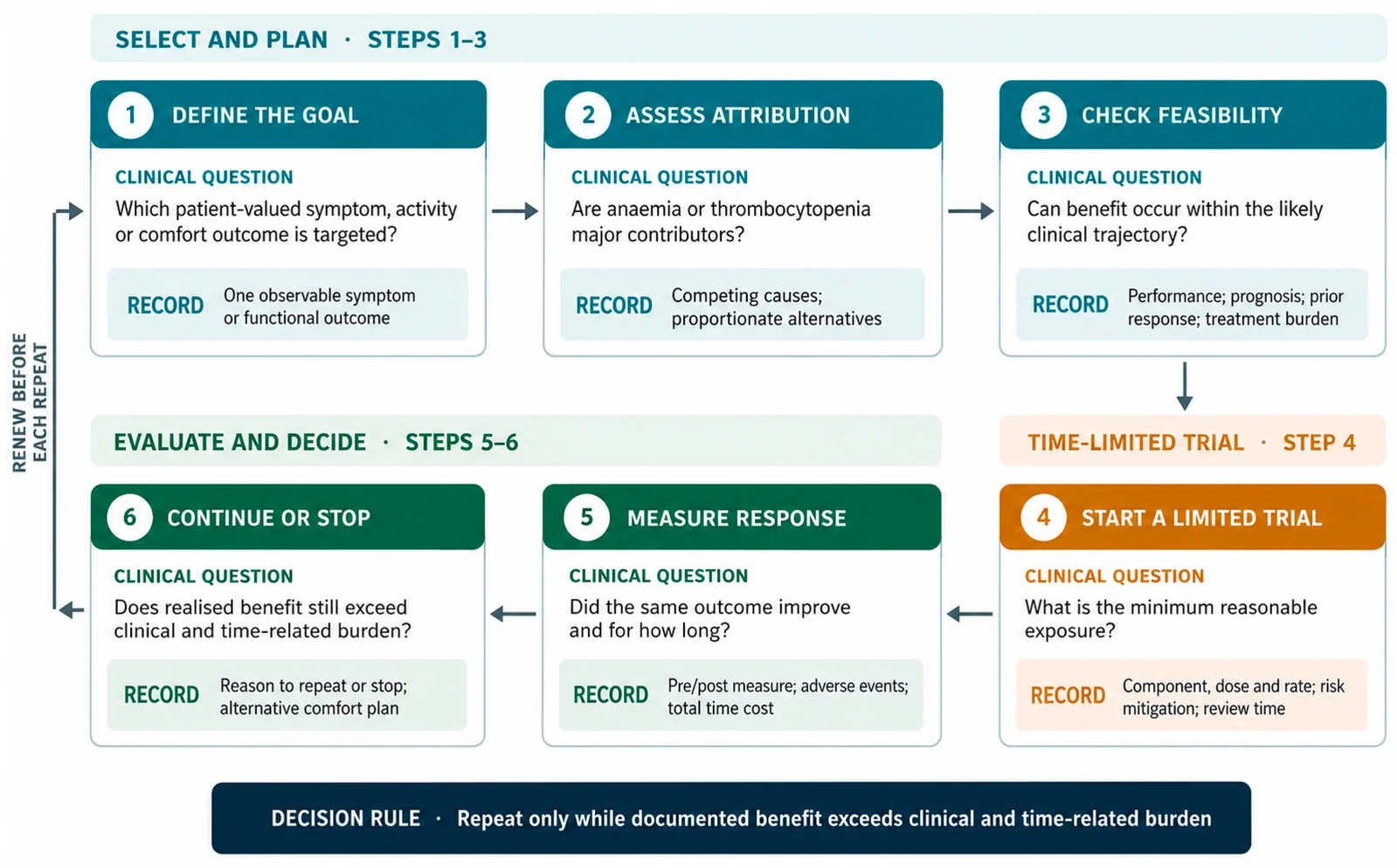
Goal-concordant, time-limited framework for transfusion near the end of life. The framework separates patient selection and planning (steps 1–3), a limited transfusion trial (step 4), and assessment and decision-making (steps 5–6). The same patient-valued outcome should be recorded before and after transfusion. Each repeat transfusion requires renewed assessment and should proceed only while documented benefit outweighs the clinical and time-related burden.

When benefit is uncertain, transfusion can be offered as a goal-concordant, time-limited trial. The patient and clinical team should define one observable goal, assess whether anaemia, thrombocytopenia or coagulopathy contributes, consider prognosis and treatment burden, and agree when response will be assessed. The fewest units likely to achieve the goal should then be given; in a stable, non-bleeding adult receiving red cells, this will usually be one unit followed by reassessment [57]. Further transfusion should require the benefit of sufficient magnitude and duration. Reasons to stop include no meaningful response, progressively shorter benefit, increasing frequency, adverse effects, excessive attendance burden or active dying. Discontinuation should be accompanied by an alternative plan for fatigue, dyspnoea, anxiety and bleeding. It changes how comfort is pursued, not the commitment to care. Figure 1 summarises this approach.

Implementation requires clear clinical responsibility. The requesting clinician should document the goal and baseline assessment; the team administering the transfusion should record the component and dose, adverse events, and time spent receiving care; and the responsible palliative-care or haematology clinician should review the response before another request is made. Advance care planning should address transfusion before a crisis, including the patient’s expectations, the outcomes that would justify continuation and the changes that would prompt discontinuation. Electronic ordering systems could require a stated palliative indication and documentation of the previous response. The audit should examine symptom response, single-unit use, frequency of repeat transfusions, adverse events, days at home, and reasons for stopping, rather than component use alone.

### 3.8. Research Priorities

Further retrospective studies describing transfusion thresholds and component use are unlikely to address the main uncertainties. Prospective pragmatic studies should compare symptom-guided with laboratory-guided transfusion strategies and assess both the immediate response and duration of benefit. Although conventional randomised trials may be difficult to conduct in end-of-life care, alternative approaches such as prospective registries, stepped-wedge studies and carefully structured individual time-limited trials could provide clinically meaningful evidence. A core outcome set should include fatigue, dyspnoea, attainment of a patient-defined goal, global impression of change, distress from bleeding, days at home, caregiver burden, adverse events and total treatment time. Studies should report non-response and attrition explicitly rather than analyse only patients who survive to follow-up.

Research into predictors of benefit should examine previous response, performance status, active bleeding, haemoglobin trajectory and the likelihood that symptoms are attributable to anaemia. Platelet studies must distinguish prophylactic from therapeutic intent. Patient blood management studies should define iron status, prognosis, symptom response and time to benefit; haemostatic studies should report transfusion-free days, re-bleeding and treatment burden. Comparisons of hospital, hospice and home transfusion require denominator-based haemovigilance, emergency-transfer rates, cost and caregiver time. Demonstrations of alternative funding models should measure goal-concordant days at home, not simply the number of transfusions delivered.

## 4. Limitations

This review is limited by both its narrative design and the underlying evidence. No randomised trial has tested red-cell transfusion for symptom relief near the end of life; most studies were retrospective, observational and single-centre. Their findings cannot be interpreted on an equal footing: prospective cohorts with prespecified assessment provide stronger evidence of symptom change than audits or chart reviews, while surveys and qualitative studies address practice or experience rather than effectiveness. Because no formal risk-of-bias or certainty assessment was undertaken, this review does not assign a graded strength to each conclusion.

Clinical heterogeneity is substantial. Studies included advanced solid tumours, acute leukaemia, myelodysplastic syndromes, haematopoietic stem-cell transplant recipients and other marrow-failure syndromes, for whom transfusion intent, disease trajectory and expected benefit differ. Outcomes were assessed by patients, physicians, nurses or caregivers using different tools and time points. High mortality and loss to follow-up mean that patients who survived and remained well enough for reassessment may be over-represented; complete-case response rates may therefore overestimate benefit. Incomplete records also cannot distinguish true non-response from failure to assess.

Survival and place-of-death comparisons are especially vulnerable to selection, confounding by indication and time-related bias. Evidence concerning platelets, plasma and cryoprecipitate is mainly descriptive. Evidence on patient blood management is indirect, and home-transfusion studies generally lack hospital comparators and use selective eligibility criteria. Although the search was broad and structured, study selection and synthesis were purposive; English-language literature was prioritised, and no meta-analysis was undertaken. Publication and selective-outcome reporting bias are likely.

## 5. Conclusions

Blood transfusion can provide meaningful, although usually temporary, palliation for selected patients receiving end-of-life care, particularly those with terminal cancer or transfusion-dependent haematological malignancies. Current evidence supports neither routine transfusion based solely on an abnormal blood count, a universal end-of-life transfusion threshold, nor automatic discontinuation because prognosis is limited. Decisions should instead consider whether transfusion is likely to relieve a defined symptom or control clinically significant bleeding within the patient’s remaining life expectancy, and whether this benefit outweighs adverse effects and treatment burden. Patient blood management may reduce the need for transfusion when a reversible cause of anaemia or a controllable source of bleeding is identified and sufficient time remains for an alternative treatment to take effect; however, it should not delay urgent relief of severe symptoms or bleeding. A central weakness in current practice is the failure to define the intended clinical outcome and reassess it after transfusion. When benefit is uncertain, transfusion should be offered as a goal-concordant, time-limited trial using the fewest units likely to achieve the agreed clinical goal. The same patient-reported symptom or functional outcome should be assessed before and after treatment, with a clinically meaningful target response and explicit stopping criteria defined in advance. Hospice and funding models should enable palliative transfusion for patients likely to benefit without making continued transfusion automatic. Future research should prioritise goal attainment, days at home, caregiver burden, bleeding-related distress and treatment time rather than blood-count increments or unadjusted survival.

## Figures and Tables

**Table 1:** Clinical effectiveness and duration of benefit after red-cell transfusion near the end of life.

Study and Population	Design and Sample	Outcome Assessment	Clinical Effectiveness and Duration
**Gleeson and Spencer, 1995** [6] UK; advanced cancer	Prospective multicentre; 97 patients transfused (91 complete records)	Patient VAS for strength, breathing and well-being; joint physician-patient dependency scale; baseline, day 2 and day 14	51/67 (76%) felt better at day 2 and 34/47 (72%) at day 14. Strength and well-being remained above baseline at day 14; 28/91 died within 14 days.
**Monti et al., 1996** [7] Italy; terminal cancer	Retrospective inpatient study; 31 patients, 35 transfusion admissions	Next-day patient-reported global well-being recorded as improved/not improved	Improvement followed 18/35 admissions (51.4%). Duration beyond the next-day assessment was not reported.
**Brown and Bennett, 2007** [12] UK; eight hospices	Multicentre survey; 164 patients, 230 episodes; Barthel data for 51 episodes	Modified Barthel Index immediately before and 1 week after transfusion; assessor not reported	Median Barthel score declined from 70 to 66, with no evidence of improved physical function. Median survival after first transfusion was 42 days.
**Laird et al., 2008** [8] UK; two palliative-care units	Observational records; 73 patients, 100 episodes	Chart documentation of a ‘positive response’; criteria and timing not defined	A response was documented for 38/100 episodes, of which 31 were positive; 10/73 patients died within 7 days.
**Martinsson and Lundstrom, 2009** [21] Sweden; specialist palliative care	Cross-sectional survey; 174/1046 patients received red cells	Physician/nurse global impression; no fixed assessment time	117/174 (68%) were judged to benefit; benefit was uncertain in 44, absent in 10 and unassessed in 3.
**Brown et al., 2010** [13] UK; cancer-related fatigue	Prospective feasibility study; 48 commenced, 30 analysable	Patient-reported FACT-F, Brief Fatigue Inventory and direct benefit question; 3 days after transfusion	21/30 achieved a clinically important FACT-F improvement and 22/30 reported less fatigue; severe fatigue fell from 27/30 to 15/30.
**To et al., 2017** [9] International; 17 palliative-care sites	Prospective cohort; 141 at baseline, 101 evaluable for day-7 benefit	Clinician-rated CTCAE grades for a nominated target symptom; baseline and day 7	The target symptom improved after 49% of evaluable transfusions and at least one symptom after 78%. Day-2 adverse effects occurred in 16/131 (12%).
**Woodwark and Dean, 2017** [22] UK; inpatient hospice	Retrospective records; 39 patients, 46 transfusions	Patient-reported benefit in narrative notes or occasional verbal symptom scores; timing varied	Benefit was documented after 18/46 transfusions; 22 had no documented benefit and 6 were missing. Half of patients died within 1 month.
**Sirianni et al., 2019** [23] Canada; two palliative-care units	Retrospective records; 24 patients	ESAS at one site and narrative target-symptom change at the other; timing varied	20/24 (83.3%) were coded as benefiting; 3 did not benefit and 1 was not assessable. Median time to death was 35 days.
**Timilsina et al., 2019** [24] Nepal; advanced solid cancer	Non-randomised study; 46 transfused and 76 non-transfused patients; allocation largely haemoglobin-based	Patient-reported presence of fatigue and breathlessness; baseline and day 7	In transfused patients, fatigue fell from 37/46 to 20/46 and breathlessness from 35/46 to 9/46. Fatigue also improved in controls; no control had baseline breathlessness.
**Neoh et al., 2019** [10] UK; national hospice audit	Prospective audit; 465 episodes at 83 hospices	Clinician judgement up to day 30; standardised patient-reported measures were not used	Improvement was sustained to day 30 after 83 episodes (18%) and lasted <14 days after 142 (31%); 50 had no improvement, 150 patients died and 40 outcomes were missing.
**Marote et al., 2022** [25] Portugal; advanced cancer	Retrospective cohort; 179 patients, 301 episodes	Chart-recorded symptomatic benefit within 15 days; no standardised scale	Benefit was recorded after 107/301 episodes (36%), no benefit after 93 and an unknown outcome after 101; benefit was 53.5% among assessed episodes.
**Ferraz-Goncalves et al., 2025** [26] Portugal; five palliative-care teams	Multicentre retrospective study; 86 patients, 122 episodes	Chart-recorded symptomatic improvement; timing not standardised	Improvement was documented after 30/122 episodes (25%); 19 had no improvement and 73 (60%) had no recorded outcome. Median survival was 15.5 days.
**Callaghan et al., 2025** [11] Saudi Arabia; palliative-care unit	Retrospective cohort; 84 patients, 159 episodes	Chart documentation; target symptom and assessment timing usually unspecified	No symptomatic improvement was documented after 82.4% of episodes. Median survival was 18 days and 83% died during the same admission.
**Miyashita et al., 2026** [50] Japan; terminal haematological or solid malignancy	Retrospective home-care study; 77 patients, 832 episodes	Patient Yes/No report of six symptoms at transfusion and the following day	Fatigue improved in 32/34 (94.1%) haematological versus 7/13 (53.8%) solid-tumour patients; breathlessness in 19/21 (90.5%) versus 3/6 (50.0%). Longer-term benefit was not measured.

**Interpretation:** Except for a non-randomised comparator study [24], the evidence is uncontrolled; many assessments were retrospective or incomplete. Response estimates are not directly comparable because populations, assessors, outcomes, timings and denominators differed. Absence of documented benefit is not equivalent to confirmed non-response. CTCAE, Common Terminology Criteria for Adverse Events; ESAS, Edmonton Symptom Assessment System; FACT-F, Functional Assessment of Cancer Therapy-Fatigue; VAS, visual analogue scale.

**Table 2:** Observed and reported red-cell and platelet transfusion practices near the end of life.

Study, Design and Population	Indication, Trigger and Dose	Reassessment and Practice	End-of-Life or Delivery Context
**Gleeson and Spencer, 1995** [6] Prospective multicentre; 97 patients	Weakness 91%, dyspnoea 41%, previous benefit 27%, bleeding 7%; median haemoglobin 79 g/L; dose not reported	Patient symptom scales at days 2 and 14; dependency assessed jointly	57% transfused in hospice and 42% in hospital; 28/91 died within 14 days
**Monti et al., 1996** [7] Retrospective; 31 patients, 35 admissions	Haemoglobin ≤ 8 g/dL and/or severe fatigue or dyspnoea; mean 2.8 units per patient	Next-day global well-being recorded as improved/not improved	14/35 admissions ended in discharge home; 21 patients died during admission
**Brown and Bennett, 2007** [12] Hospice survey; 164 patients, 230 episodes	Two units in 39% and three in 37% of episodes; mean haemoglobin 81 g/L in the detailed hospice subset	Barthel score reassessed at 1 week for 51 episodes	83% of episodes were inpatient; median survival after first transfusion was 42 days
**Laird et al., 2008** [8] Observational records; 73 patients, 100 episodes	Low haemoglobin in 73% and sole indication in 26%; mean haemoglobin 79 g/L; mean 2.2 units	Response recorded after only 38% of episodes; patient/family discussion undocumented in 78%	Two UK specialist palliative-care inpatient units; most patients were in their final two months of life. Mean interval from last transfusion to death: 47 days; 13.7% died within 7 days
**Martinsson and Lundstrom, 2009** [21] Cross-sectional survey; 174 transfused	Mean 2.9 units over 1.6 occasions; predefined trigger in 103 patients (mean approximately 90 g/L)	Staff global impression; 68% judged to benefit	82/141 home-care patients received transfusion at home
**Goksu et al., 2014** [28] Final-admission cohort; 398 solid-tumour patients	Red cells: low haemoglobin ± symptoms 73.9%, bleeding 26.1%, mean 4.37 units; platelets: prophylaxis 77.5%, bleeding 22.5%	Symptom response was not assessed	Hospital medical-oncology service without dedicated palliative-care unit; Median final admission: 15 versus 8 days for red-cell recipients/non-recipients and 16 versus 10 days for platelet recipients/non-recipients
**Woodwark and Dean, 2017** [22] Retrospective hospice study; 39 patients, 46 transfusions	Fatigue 84%, dyspnoea 41%; mean haemoglobin 76.1 g/L; mean 2.5 units	Narrative reassessment was inconsistent; 6 outcomes missing	UK inpatient hospice; all transfusions were administered in the hospice, including 11 patients admitted from home solely for transfusion. 50% died within one month
**Wang et al., 2018** [32] Retrospective; 116 deceased HSCT recipients	Mean red cell use 0.7 units with or without hospice; platelet use 0.8 versus 1.2 units with versus without hospice	No symptom, bleeding or quality-of-life reassessment	Without hospice, 17% were transfused on the day of death and 31% the day before; 75% with hospice had last transfusion >96 h before death
**Chin-Yee et al., 2019** [14] Physician survey; 29 respondents	Symptoms 97%, bleeding 62%, low haemoglobin 52%; 13/15 threshold users selected <70 g/L	All reported reassessment, but 72% used no objective symptom scale	Hospital attendance was considered a barrier by 90%; 89% judged the evidence weak
**Sirianni et al., 2019** [23] Retrospective; 24 patients	Fatigue 83.3%, dyspnoea 25%; median haemoglobin 65 g/L; 83.3% received one unit	Target symptom documented; ESAS or narrative reassessment; further transfusion withheld if no benefit	Median time to death was 35 days Two hospital-based inpatient palliative-care units for transfusion
**Timilsina et al., 2019** [24] Non-randomised; 46 transfused	All patients with haemoglobin <10 g/dL were transfused; mean 1.36 units and 69.5% received one unit	Fatigue and breathlessness reassessed at day 7	Inpatient and outpatient settings combined; follow-up ended at day 7
**Neoh et al., 2019** [10] National hospice audit; 465 episodes	Mean haemoglobin 75 g/L; low haemoglobin alone 51%, with dyspnoea 40%, fatigue 16%; 84% received ≥ 2 units	Haemoglobin rechecked in 28%, performance status in 14%, and weight recorded in 15%	65% occurred during hospice admission; 32% died within 30 days
**Moracchini et al., 2021** [15] Retrospective; 119 haematology patients, 1999 platelet concentrates	55% prophylactic; 18.3% given in the final 2 weeks and 33.5% in the final month	Bleeding burden increased, while platelet recovery and transfusion interval declined near death	French university-hospital haematology service, involving inpatient or single-day attendance. 7 patients died from grade-4 haemorrhage; 5 were transfuse for the fatal event
**Brome Uribe et al., 2021** [30] Retrospective; 151 patients in final 6 months Multicentre institutional cohort in Medellín	Indications and triggers not reported. Patients received: Red cells (*n = 124*), platelets (*n = 17*), plasma (*n = 8*) and cryoprecipitate (*n = 3*); categories overlapped. Median dose: 1 unit per patient (IQR 1–2).	Symptoms before/after first and last transfusion were incompletely documented	Adult/paediatric and inpatient/outpatient settings combined
**Marote et al., 2022** [25] Retrospective; 179 patients, 301 episodes	Mean haemoglobin 6.85 g/dL; fatigue/asthenia common; bleeding 33%; median one unit per episode	Symptoms recorded before 67% of episodes; benefit unknown after 34%	Portuguese oncology palliative-care service; 58% of episodes involved inpatients and 42% involved outpatients seen in emergency or clinic settings. Median time from transfusion to death: 41 days; 30-day survival 57%
**Ostan et al., 2024** [27] Home-care cohort; 1108 patients, 179 transfused	Mean haemoglobin before first transfusion approximately 7.4 g/dL in solid and 7.6 g/dL in haematological malignancy	No post-transfusion symptom or haemoglobin outcomes	Transition at home through specialist palliative-care programme in Bologna. 52% received the last transfusion within 30 days of death, within 7 days in 20.6% and 5% within 2 days
**Ferraz-Goncalves et al., 2025** [26] Five Portuguese palliative-care teams, retrospective; 86 patients, 122 episodes	Median haemoglobin 7.4 g/dL; fatigue 38%, low haemoglobin alone 37%, bleeding 33%; dose not reported	Improvement documented after 25%, none after 16%, and no outcome after 60%	68% of episodes occurred in inpatient palliative care and 32% in other hospital departments. No transfusions were administered at home; median survival after last transfusion was 15.5 days, volume overload in 3%
**Callaghan et al., 2025** [11] Retrospective; 84 patients, 159 episodes	Low haemoglobin in 95%; mean 64.5 g/L; one unit 47.2%, two units 52.2%	No improvement documented after 82.4% of episodes	Transfusion at specialised hospital inpatient palliative-care unit; Median survival 18 days; 83% died during same admission
**Miyashita et al., 2026** [50] Home-care study; 77 patients, 832 episodes	Usual trigger 60–70 g/L, modified for symptoms/comorbidity; two units per episode	Vital-sign monitoring and next-day symptom review; transfusion stopped when benefit ceased	All transfusions at home; median interval from last transfusion to death 4.4 days in haematological and 18.1 days in solid malignancy
**Hattori et al., 2026** [33] Hypothetical physician survey; 596 respondents	Symptomatic threshold: ≤8.0 g/dL for haematologists and ≤7.0 g/dL for palliative-care physicians; lower/variable thresholds when asymptomatic	Most supported reducing frequency (76.1% and 71.1%), but few supported discontinuation (9.3% and 19.7%)	Reported decisions only; no observed transfusions or outcomes

**Interpretation**: Findings are descriptive and should not be treated as comparative effectiveness estimates. Hattori et al. [33] report decisions in hypothetical scenarios rather than observed care. HSCT, haematopoietic stem-cell transplantation.

**Table 3:** Patient blood management evidence relevant to transfusion avoidance near the end of life.

Study	Population	Intervention	Relevant Finding	Interpretive Limitation
Dickson et al., 2023 [38]	Randomised feasibility trial; 34 advanced-cancer patients	Intravenous iron vs. placebo	Anaemia resolved at 8 weeks in 39% compared with 8%; fatigue scores favoured iron, with imprecision.	Underpowered for efficacy or transfusion reduction; performance status 0–2; active bleeding excluded.
Yeung et al., 2026 [39]	Retrospective cohort; 283 evaluable IV-iron episodes	Intravenous iron	Haemoglobin increased by day 28; red cell use decreased, including during supportive care.	No control group or symptom outcomes; patients who died before day 28 were excluded.
Ng et al., 2018 [40]	Feasibility trial; 27 patients receiving palliative chemotherapy	Intravenous iron isomaltoside during palliative chemotherapy	Possible quality-of-life benefit; no significant difference in haemoglobin or transfusion use.	Small, baseline imbalance; not an end-of-life cohort.
Saito et al., 2022 [41]	Prospective multicentre cohort; 55 bleeding gastric cancers;	Palliative radiotherapy (RT)	Four-week response 53% by intention to treat and 78% per protocol.	No comparator; early deaths strongly affected response estimates.
Mitsuhashi et al., 2021 [42]	Retrospective gastric-cancer cohort; 28 patients; palliative RT	Palliative RT	No patient required transfusion within one month; six were later transfused at a median of 99.5 days.	Heterogeneous treatment; three died within one month; one grade-5 bleed.
Katano and Yamashita, 2023 [43]	Retrospective mixed-tumour cohort; 21 transfusion-dependent patients	Palliative RT	19 required fewer red-cell units; 14 transfusion-free for 30 days.	Small, heterogeneous sites and fractionation; no comparator.
Yamada et al., 2023 [44]	Three end-of-life solid-tumour cases with enhanced-fibrinolytic disseminated intravascular coagulation	Heparin plus tranexamic acid	Bleeding resolved; platelet/plasma support stopped in two cases.	Case evidence only; one cerebral infarction; not generalisable to other forms of disseminated intravascular coagulation.

## Data Availability

No new data were created or analyzed in this study. Data sharing is not applicable to this article.
